# Design and Development of a Viral Hepatitis and HIV Infection Screening Program (Hprolipsis) for the General, Greek Roma, and Migrant Populations of Greece: Protocol for Three Cross-Sectional Health Examination Surveys

**DOI:** 10.2196/13578

**Published:** 2020-01-31

**Authors:** Giota Touloumi, Argiro Karakosta, Vana Sypsa, Ioanna Petraki, Olga Anagnostou, Agis Terzidis, Niki Maria Voudouri, Doctors Of The World Greek Delegation, Magda Gavana, Apostolos Vantarakis, George Rachiotis, Maria Kantzanou, Theofilos Rosenberg, George Papatheodoridis, Angelos Hatzakis, Hprolipsis Study Group

**Affiliations:** 1 Department of Hygiene, Epidemiology, and Medical Statistics Medical School National and Kapodistrian University of Athens Athens Greece; 2 International Medicine-Health Crisis Management School of Medicine National and Kapodistrian University of Athens Athens Greece; 3 Programs of Development, Social Support, and Medical Cooperation (PRAKSIS) Athens Greece; 4 see Acknowledgments; 5 Department of Primary Health Care, General Practice, and Health Services Research Medical School of Aristotle University Thessaloniki Greece; 6 Environmental Microbiology Unit of Public Health Medical School University of Patras Patra Greece; 7 Department of Hygiene and Epidemiology Medical Faculty University of Thessaly Larisa Greece; 8 Department of Gastroenterology, Laiko General Hospital School of Medicine National and Kapodistrian University of Athens Athens Greece

**Keywords:** hepatitis, HIV, Greek general population, Greek Roma, migrants, health examination surveys

## Abstract

**Background:**

Although infectious diseases are globally on the decline, they remain a major global public health problem. Among them, the hepatitis B virus (HBV) or hepatitis C virus (HCV) and HIV infection are of primary interest. Valid prevalence data on these infections are sparse in Greece, especially for vulnerable populations.

**Objective:**

This study aimed to present the design and methods of Hprolipsis, an integrated viral hepatitis and HIV screening program administered to adults (≥18 years) from the general, Greek Roma, and migrant populations. Its aims were to estimate the prevalence of HBV, HCV, and HIV; assess infectious disease knowledge level; design, implement, and assess population-specific awareness actions; and offer individual counseling and referral when indicated and HBV vaccination to susceptible Roma and migrants.

**Methods:**

Multistage, stratified, random sampling based on the 2011 Census was applied to select the general population sample, and nonprobability multistage quota sampling was used for Roma and migrant sample selection. Trained personnel made home (general population) or community (Roma and migrants) visits. Collected blood samples were tested for Hepatitis B surface Antigen, Hepatitis B core Antibody, Hepatitis B surface Antibody, Hepatitis C Antibody, and HIV 1,2 Antibody. The surveys were conducted during May 2013 and June 2016. To estimate an HCV prevalence of 1.5% with 0.3 precision, the required general population sample size was estimated to be 6000. As migrants constitute 10% of the whole Greek population, the migrant sample size was set to 600. A feasible sample size of 500 Greek Roma was set.

**Results:**

In total, 6006 individuals from the general population (response rate 72%), 534 Greek Roma, and 612 migrants were recruited. Blood test results are available for 4245 individuals from the general population, 523 Roma, and 537 migrants.

**Conclusions:**

Hprolipsis is the first nationwide survey on HBV, HCV, and HIV. Its results will enhance our understanding of the health needs and disease burden of these diseases in the 3 studied populations. Its implementation provided useful recommendations for future studies, particularly in vulnerable populations.

**International Registered Report Identifier (IRRID):**

DERR1-10.2196/13578

## Introduction

### Background

Although infectious diseases are on the decline globally, they are still of major public health importance, imposing significant burden on global economies and public health [1]. Among them, hepatitis B virus (HBV) or hepatitis C virus (HCV) and HIV infection are of particular interest in developed countries owing to their prevalence and associated disease burden.

In 2015, about 257 million people were living with chronic HBV infection and 71 million people with chronic HCV infection globally [2]. An estimated 36.7 million people worldwide were living with HIV at the end of 2015 [3]. Currently, highly effective therapies for HCV and long-term therapies for HBV and HIV infections are available. However, owing to their usually long asymptomatic period, only a minority of those infected with one of these viruses are aware of their infection, lacking thus the opportunity to take advantage of new treatments [4]. Delayed diagnosis has harmful implications not only for individual patients but also for public health owing to the ongoing viral transmission from undiagnosed incidents. The World Health Assembly, in 2016, introduced a global strategy for achieving viral hepatitis elimination by 2030 (reduce incidence by 90% and mortality by 65%) [5]. In addition, the Joint United Nations Programme on HIV and AIDS targets to curb the HIV epidemic by 2020, including diagnosis of 90% of those living with HIV, treatment of 90% of those diagnosed, and achieving viral suppression of 90% of those treated [6]. Valid estimates on prevalence, infection awareness, treatment uptake and the knowledge for HBV, HCV, and HIV are necessary to plan and implement effective prevention programs.

In Greece, until recently, estimates for HBV and HCV prevalence were derived from studies restricted to specific population groups (eg, patients undergoing hemodialysis, people who inject drugs, men who have sex with men, and blood donors) [7-11]. In 2015, a telephone survey was conducted in a representative sample of adults living in Greece. On the basis of self-reports, the (age-adjusted) prevalence (95% CI) of chronic HBV and HCV was estimated as 2.39% (1.88%-2.91%) and 1.79% (0.97%-2.61%), respectively [12]. Trends in HIV diagnosis are reported by the HIV/AIDS surveillance system operated by the Hellenic Centre for Disease Control and Prevention (HCDCP), but delayed diagnosis, duplicates, or missing information on key characteristics of the infected population cannot be ruled out [13].

Vulnerable populations, such as the Greek Roma and migrants, constitute special target groups since their adverse living conditions facilitate the spread of infections. Although data on Roma health are sparse and fragmentary, Roma communities appear to have unequal access to health services, high morbidity, especially regarding infectious diseases, and insufficient immunization compared with that of the general population [14,15]. The combination of increased risk factors for infectious diseases and lack of appropriate models for reaching, engaging, and informing Roma communities raises serious public health concerns [16,17]. In a recent study conducted in Slovakia, it was found that the Roma population has a higher prevalence of HBV compared with non-Roma populations [18]. In Greece, only a small proportion of Greek Roma is integrated into mainstream society, whereas the vast majority lives marginalized on the outskirts of inhabited areas [19]. The few available studies on Roma health have shown that they have been experiencing a higher prevalence of infectious diseases [20], lower vaccination coverage [21,22], and higher relative mortality from infectious diseases compared to non-Roma Greeks [23].

Migrants are another population group with increased incidence of infectious diseases. In Europe, migrants accounted for 40% of newly reported HIV cases between 2007 and 2011 [24]. The migration process itself increases the risk of infectious diseases [3]. Migrants are more likely to be exposed to social factors associated with poor health such as poverty and unemployment; they may face social exclusion, whereas legal and administrative issues may pose additional barriers to access to health services [3,25]. It has been shown that migrants infected with infectious disease are diagnosed later than native populations, limiting their treatment options and increasing the probability of transmission [3,25,26]. In Greece, data on viral hepatitis among migrants are limited; only new HIV diagnoses are reported to the HIV/AIDS surveillance system of the HCDCP.

Taken together, accurate data on the prevalence, awareness rates, treatment uptake, and knowledge related to HBV, HCV, and HIV in the general population and especially among specific vulnerable populations are currently lacking in Greece. To fill this gap, the Hprolipsis Survey, a Design and Development of Viral Hepatitis and HIV Infection Screening Program in the General Population and Vulnerable Populations, was set up. Hprolipsis, combining questionnaire data on health determinants, living conditions, high-risk behaviors, and knowledge on viral hepatitis and HIV with results from serological testing, provides a unique opportunity to study HBV, HCV, and HIV. It focused on 3 target populations: adult general population, Greek Roma, and migrants. Experience gained through Hprolipsis implementation could provide valid guidelines for future studies. In this paper, we describe the design and methodology of Hprolipsis, and we discuss the challenges associated with organizing and implementing such a study focusing on difficult to reach populations, such as the Greek Roma and migrants.

### Objectives of the Study

This study aimed to assess access to the public health care system; estimate the prevalence and determinants of HBV, HCV, and HIV infection in the 3 target populations; design and implement population-specific awareness-raising actions; offer anti-HBV vaccination to susceptible Greek Roma and migrants study participants; and offer individual counseling and referral to health care services for all individuals who were tested positive for any of these infections.

The specific objectives of the study were as follows:

1. To estimate HBV, HCV, and HIV prevalence and cascade of treatment in the 3 studied populations.
2. To assess knowledge level and effectiveness of the awareness-raising activities.
3. To investigate barriers and facilitators to access to care.
4. To provide counseling—referral to individuals who tested positive for any of these infection.
5. To offer HBV vaccination to susceptible individuals in vulnerable populations.

## Methods

### Study Design

Hprolipsis consists of 3 cross-sectional epidemiological surveys of 3 adult (≥18 years) populations: (1) the general population, (2) Greek Roma, and (3) migrants. It was funded by the European Union’s (EU) structural funds and national resources, coordinated by the Department of Hygiene, Epidemiology, and Medical Statistics of the Medical School of the National and Kapodistrian University of Athens (NKUA), and conducted in cooperation with all the other Greek medical schools, the MSc International Medicine—Health Crisis Management of the Medical School of NKUA, and the nongovernmental organizations (NGOs) Doctors of the World Greek delegation and PRAKSIS. Hprolipsis was initiated in May 2013 and completed in June 2016. The general population survey was nested within the National Survey of Morbidity and Risk Factors (EMENO) health examination survey; EMENO aimed to investigate morbidity (focused on cardiovascular and respiratory diseases) and associated risk factors in the general adult population, which have been described in detail elsewhere [27].

### Sampling Strategy

For the general population, a multistage stratified random sampling based on the 2011 Census was applied (for a detailed description of the sampling strategy, refer to the study by Nikolaidis et al [27]); the target sample size was 6000 individuals, allowing us to estimate a 1.5% prevalence of anti-HCV with 0.3% precision [23]. Nonprobability multistage quota sampling was applied to select Greek Roma and migrant populations.

#### Greek Roma Population

In the Greek national context, the Roma are Greek citizens who are not officially recognized as a national or linguistic minority. Thus, little reliable data about the Roma in Greece have been collected. Estimates of Roma populations in Greece range from 120,000 to 300,000 [22]. Greek Roma populations live mainly in 4 settlement types: (1) houses dispersed among the majority population, (2) settlements with houses, (3) settlement/camps with houses and shacks (mixed), and (4) shacks. For the purposes of this program, those living in houses dispersed among the majority population were excluded as they would be part of the general population survey. Balancing the available financial resources and feasibility/difficulty of running a health survey in Greek Roma settlements, the target sample size was set at 500 adults. To increase the representativeness of the sample, a 3-stage procedure was adopted.

#### Stage 1

An effort was made to collect and synthesize information on Greek Roma settlements by type of settlement and relevant population, and general living conditions in the camps. Data were collected from the Ministry of Interior, Greek Roma organizations, and the relevant literature.

#### Stage 2

On the basis of data retrieved from stage 1, 4 geographical regions were selected: Peloponnese (Western Greece), Thessaly (Central Greece), and Central Macedonia (Northern Greece) as regions with higher Greek Roma numbers [17], and Attica as the largest urban center in Greece. In each selected geographical region, areas representative of the 3 settlement types (houses, mixed, and shacks) were selected. Selected settlements were visited by specialists (social and/or health scientists), and the population demographics as well as the general living conditions in each selected settlement were recorded.

#### Stage 3

To increase the power of investigating potential differences by geographical region, we recruited the same number of people in each region (135 adults per region). Within each region, the recruited number of people from each type of settlement was broadly proportional to its size (Table 1) as recorded by the study staff (stage 2). Adults (≥18 years) from the selected settlements were invited to participate in the study. Only 1 adult from each family could participate. An effort was made to reflect in our sample the actual sex and age distribution of the settlement as recorded in stage 2 (ie, the required number of men and women by 10-year age groups was predefined).

**Table 1:** Planned and achieved Greek Roma population sample per region participating in the study and settlement type.

Selected settlements and settlement type		Houses	Families per household	Persons per household	Planned sample	Final achieved sample
**Attica**						
	ST^a^ 1^b^: Acharnes	300	2 to 3	5 to 10	45	45
	ST 2^c^: Ano Liosia	300	2 to 4	5 to 8	45	43
	ST 3^d^: Aspropyrgos	200 to 300	2	8	45	46
Region total		800 to 900	2 to 4	5 to 10	135	134
**Western Greece**						
	ST 1: Kato Achaya	2000	1	9	95	93
	ST 2: Sagianeika	150	3	20	30	20
	ST 3: Riganokampos	20	3	15	10	30
Region total		2170	1 to 3	9 to 15	135	143
**Thessaly**						
	ST 1: Nea Smyrni	400	1 to 3	5 to 10	53	54
	ST 2: Tyrnavos	400	1 to 3	5 to 8	53	54
	ST 3: Farsala	150 to 200	1 to 3	5 to 10	29	30
Region total		950 to 1000	1 to 3	5 to 10	135	138
**Central Macedonia**						
	ST 1: Dendropotamos	500	2 to 3	6 to 10	45	36
	ST 2: Agia Sofia	300	2 to 3	8 to 10	45	42
	ST 3: Peraia	65 to 70	1 to 2	4 to 5	30	26
	ST 3: Chalastra	20	1 to 2	5 to 8	15	15
Region total		885 to 890	1 to 3	4 to 10	135	119
Total		4805 to 4960	1 to 4	4 to 10	540	534

^a^ST: settlement type.

^b^ST 1: settlement with houses.

^c^ST 2: settlement with houses and shacks (mixed).

^d^ST 3: settlement with shacks.

#### Migrant Population

Lack of reliable data on the composition of migrants and their high mobility during the study period made it very difficult to select a random sample. To reduce the sampling error, it is recommended to approach migrants through multiple sources [22,28].

Migrants were defined as those who were born in another country. Excluded were those who originated from the EU-15 countries (Austria, Belgium, Denmark, Finland, France, Germany, Greece, Ireland, Italy, Luxembourg, the Netherlands, Portugal, Spain, Sweden, and United Kingdom), Norway, Switzerland, the United States, and Canada. Inclusion criteria were (1) aged 18 years or older at recruitment and (2) living in Greece for at least 6 months (self-reported); thus, new arrivals and/or those living in shelter camps were excluded to avoid confusing data on health care access. To select the sample, a 2-stage procedure was followed.

#### Stage 1: Preparation Phase

All available data from the Ministry of Interior, Asylum Service of the Ministry of Migration Policy, relevant literature, as well as data from the Hellenic Statistical Authority (ELSTAT) were reviewed. As undocumented migrants are likely to be unrepresented in the Ministry of Interior and Asylum Service of the Ministry of Migration Policy datasets, the most reliable data were considered those reported by the ELSTAT based on the 2011 Census. According to that census, the number of those born abroad was 911,929 persons, thus consisting of approximately 10% of the total population living in Greece. To keep the sampling fraction similar to that in the general population (about 10% of the total population), the required sample size was set as 600 adults. The distribution of the region of origin of migrants based on the 2011 Census is presented in Table 2. Most migrants originated from Albania. Albanians migrated to Greece in late 90s to early 2000s, and currently most of them are well integrated into Greek society; thus, they are expected to be included in the general population survey. As this survey focused on the most vulnerable populations, it was decided to underrepresent those who originated from Albania (planned percentage 33.0% vs 54.7% in the population of migrants in Greece) and overrepresent those from regions more likely to be part of current migration flows, namely those originated from Africa and Asia (planned percentage 46.0% vs 21.0% in the population of migrants in Greece). The planned sample and the final achieved sample by region of origin are presented in Table 2.

**Table 2:** Planned and achieved migrant population sample according to country of birth.

Description	Proportion in the total adult population based on 2011 Census (%)	Planned number and proportion in the sample, n (%)	Achieved number and proportion in the final sample, n (%)
Country’s total migrants^a^	100.0	600 (100.0)	612 (100.0)
Countries members of EU^b^	17.4	90 (15.0)	67 (10.9)
Non-EU European countries (except Albania)^c^	6.4	36 (6.0)	53 (8.7)
Albania	54.7	198 (33.0)	159 (26.0)
Africa	3.2	90 (15.0)	145 (23.7)
Asia	17.8	186 (31.0)	187 (30.5)
Other (Caribbean, South or Central America, Oceania)	0.5	N/A^d^	1 (0.2)

^a^According to our inclusion criteria: adults (≥18 years), excluded those who originated from the EU-15 countries, Cyprus, Norway, Switzerland, the United States and Canada.

^b^EU: European Union.

^c^According to our inclusion criteria, those who originated from Norway and Switzerland were not included.

^d^N/A: not applicable.

#### Stage 2: Recruitment of Participants

A comprehensive mapping of NGOs and refugee and migrant communities was made. A network of NGOs and especially of migrant communities was set up, and the survey’s aims were disseminated to them. To implement the migrants’ survey, an official collaboration with the 2 largest NGOs serving migrants in Greece was set up: Doctors of the World Greek delegation and PRAKSIS. These NGOs run open polyclinics in 4 large cities/regions in Greece where the majority of migrants live: Athens-Attica (Doctors of the World and PRAKSIS), Patra-Peloponnese, Western Greece (Doctors of the World), Thessaloniki-Central Macedonia, Northern Greece (Doctors of the World and PRAKSIS) and Chania-Crete (Doctors of the World), and Southern Greece (Doctors of the World). Eligible migrants attending the polyclinics were invited to participate in the study. As those attending polyclinics may have different characteristics from those in the reference population, eligible migrants were also invited through their communities to visit polyclinics on specific days and time.

### Questionnaire Development and Measurements

A steering committee (SC) consisting of experts in epidemiology, medical statistics, virology, internal medicine, hepatology, and the representatives of the collaborating NGOs, migrants, and Greek Roma communities was set up to develop survey instruments. For the questionnaire, items previously validated were used, whenever possible. For that, an extensive systematic literature review was conducted. New questions were formulated by the SC. Two different questionnaires were created, for interviewers and physicians, with 3 different variations for the 3 target populations.

The Hprolipsis interviewers’ questionnaire involved the following sections: (1) basic demographics, (2) socioeconomic situation, (3) addictive substances usage (smoking habits, alcohol consumption, and drug use), (4) medical history, (5) eating habits focusing on Mediterranean diet adherence, (6) a short 4-item questionnaire for evaluating symptoms of anxiety and depression, (7) use and access to public health services, (8) knowledge assessment of HBV, HCV, and HIV/AIDS, (9) sexual behavior, and (10) risky behaviors related to infectious diseases. A detailed description of questionnaire items can be found in Multimedia Appendix 1. The interview mode adopted was a computer-assisted personal interview, aided by self-administered laminated cards for the most sensitive questions (eg, sexual behavior). The vast majority of the questions were close ended. All items were administered to all 3 study populations (apart from the anxiety and depression questionnaire that was not included in the migrants’ survey). Nevertheless, verbal modifications were made to adapt to each population’s culture. In addition, population-specific questions were added to the Greek Roma and migrant questionnaires; these were related to living conditions or legal status (for migrants). An additional settlement-specific short questionnaire on the status of the settlement (distance from main roads, water, and electricity supply, etc) was also developed and administered to Greek Roma local representatives.

The physicians’ questionnaire included general information about blood sampling procedure (visit outcome, place, and duration) and specific exclusion for blood sample draw criteria. Collected blood samples were tested for the following serological markers anti-HIV-1/2, anti-HCV, HBsAg, anti-HBc, and anti-HBs. Study standardized operating procedures (SOPs) were developed containing information on health surveys, sampling procedure, interview techniques, detailed guidelines on filling each questionnaire item, and collection and management of blood samples.

Survey questionnaires were tested and validated (time, clarity of questions, comprehensibility, etc) in 30 volunteers from the general population [27], 4 Greek Roma volunteers, and 4 migrant volunteers from each cooperating NGO. Feedback from questionnaire testing led to minor modifications/corrections where necessary.

### Ethics

Hprolipsis was approved by the Ethics Committee of the NKUA (date: March 4, 2015, protocol: 6141); the general population EMENO survey was approved by the same ethics committee (date: November 8, 2012, protocol: 1742) and by the Hellenic Data Protection Authority (date: December 7, 2012, protocol ΓΝ/ΕΞ/1069-1/07-12-2012).

All participants were given the time to carefully read the participant information and consent forms (PICF) and to ask relevant questions before they signed it. For Greek Roma participants, the PICF was linguistically simplified; usually, interviewers read it to the eligible adults. Cultural mediators were available, too. The migrants’ PICF was available in 9 languages (Greek, English, French, Albanian, Romanian, Farsi, Urdu, Arabic, and Bengali). An interpreter was available, when necessary. Consent from migrants was provided by ticking the corresponding icon in the computer-assisted personal interview questionnaire. Consent was confirmed in writing by the physician and another person (NGO staff or study interviewer or migrant community representative or interpreter) during the signature procedure. This procedure was adopted to avoid paper-signed PICF being a potential barrier to participation, particularly for undocumented migrants [28].

A combination of digits demonstrating the population, region, interviewer, and serial number of the participant were used to prepare barcodes with unique individual codes. Barcodes were attached to all forms and questionnaires, referral forms for blood examinations, and aliquots. For aliquots, specific deep-freeze barcodes were used. Personal data and their associated individual code were safely stored in a separate file. Only physicians and coordinators of each population/area had access to these data which were used only to send thank-you letters, medical reports, and to facilitate linkage to the public health care system when needed.

Before any field work began, all researchers and physicians signed a confidentiality form, and prophylactic vaccination against HBV was recommended.

### Implementation of the Study

#### Researchers’ Training

Training materials on data collection through questionnaire, sampling, and blood collection were developed by the SC. All training materials were available on an electronic database, accessible by all researchers. For the general population, a 2-day training program was organized on September 12 and 13, 2013 [27]. For the Greek Roma and migrant populations, 2 training seminars took place on November 11, 2014, and October 23, 2014, respectively. All study staff (coordinators, interviewers, physicians, and intercultural mediators), as well as collaborating NGOs and Greek Roma and migrant communities’ representatives participated in the course. The training course included information on the purpose and objectives of the study; the study design; portable computer usage; instructions on how to fill out questionnaires; and guidelines for blood sampling, storage, and administration of blood samples.

#### Information System

A total of 2 database-driven Web applications were developed for each population. One application was installed on each interviewer’s laptop (for the general and Greek Roma populations) or tablet (for the migrant population), whereas the other one resided on a private server. The central databases were hosted on the central server, located at the Department of Hygiene, Epidemiology, and Medical Statistics of the Medical School of the NKUA. To achieve secure access and use of data, each database on the laptops/tablets was synchronized with each database on the central server over a private network, and local data (from laptops/tablets) were eventually deleted. Personal data (names and contact details of the participants) were saved in a separate file of the database, and the participant’s code was used to connect all available information.

All data-related operations were made through secure transactions encrypted with 128-bit encryption, and compliance to atomicity, consistency, isolation, and durability was maintained in both databases of each population to ensure that all previously described operations were consistent, isolated, and durable.

### Field Study Implementation

#### General Population

The principal investigator of each collaborating medical school was responsible for the field study in the nearby regions. The whole study was piloted in a sample of 160 adults from urban and rural areas, and study materials were adjusted when necessary. Study aims were disseminated through several means including central and local press conferences, advertisement in newspapers and on TV, informing meetings with local authorities, events in central locations near the sampling points, social media, and through the Hprolipsis website [29].

A letter was sent to the local police stations about the study procedure, and a notification letter was left in each house if the owner was not present at the researcher’s visit. Following study standardized operating procedures, an adult was randomly chosen from all adults living in eligible households (ie, the one with the most recent birthday). After providing signed inform consent, an interview took place in the participants’ houses during the scheduled appointments. Following the interview, physicians made house visits for blood sample collection. In some rural areas, blood sample collection took place at nearby health centers. A separate informed consent was required for testing blood for viral HBV and HBC and for testing for HIV. A more detailed description of the field study implementation in the general population is provided elsewhere [27].

#### Greek Roma

To reach Greek Roma populations, the most important step was to develop trust relationships. To do so, several steps, inspired by the document “A practical guide for implementing health promotion activities with the Roma people [30],” were taken: (1) cultural mediators, preferably living in the selected settlements, were recruited; in most settlements, 2 mediators (1 man and 1 woman) were employed to ensure communication with both male and female participants; (2) initial visits with Greek Roma community leaders facilitated by the cultural mediators were organized in the selected settlements to inform them about the study and its aims; (3) health care services provision in the involved communities, such as dental, pediatric, and ophthalmological preventive examinations were organized before and during the field study, in collaboration with various governmental organizations and NGOs (The Smile of the Child, Therapy Center for Dependent Individuals [KETHEA], Exelixis, Athens University School of Dentistry, etc); and (4) in collaboration with The Smile of the Child, children’s clothes and toys were distributed.

Special information leaflets based on pictures and using simplified language were printed and distributed in the participating settlements to inform the community about the study aims and procedures. In collaboration with cultural mediators and Greek Roma community representatives, appropriate private places for interviewing and blood sample collection were identified in each selected settlement. Trained interviewers and physicians along with the cultural mediators visited the settlements several times, and cultural mediators invited people to participate. An effort to keep the predefined age and gender distribution of the participants was made. Physicians provided additional medical advice if requested.

#### Migrants

Initially, migrant communities and NGOs working with migrants were informed about the survey. Informative material was developed in 3 languages (Greek, English, and French) and was disseminated at the offices of the Doctors of the World Greek delegation and PRAKSIS, as well as in migrant communities and other relevant NGOs. Several events were organized in collaboration with migrant communities to further disseminate study aims and procedures. Social media was also used. In each polyclinic, an isolated office was chosen for interviewing and blood sample collection. Study interviewers and physicians visited polyclinics on prespecified dates and times and invited migrants to participate. In certain cases, interviewing took place in migrant communities’ offices. In such cases, an appointment was arranged in the nearby polyclinic for blood sample collection. To facilitate communication with potential participants, interpreters were employed.

### Blood Sample Management

Blood sampling protocol determined the procedure of collection and preservation of blood samples. Blood samples were kept in a cold environment (at 4°C) until transported, within 12 to 18 hours, to collaborating local laboratories for centrifugation. Centrifuged samples were stored at –80°C. All samples were sent to the central laboratory (National Retrovirus Reference Center, Department of Hygiene, Epidemiology, and Medical Statistics of the Medical School of the NKUA) for analysis.

### Medical Notification/Counseling

To general population survey participants who tested negative for HBsAg, anti-HCV, and anti-HIV-1/2, a thank-you letter and a copy of their blood test results along with the physician’s report (with corresponding recommendations) based on their personal medical findings were mailed. Participants who were notified if they had acquired or vaccine-induced anti-HBV immunity. Individuals susceptible to HBV were advised to be vaccinated. Participants who tested positive for HBsAg and/or anti-HCV and/or HIV-1/2 were contacted via telephone by a specialized physician. Individual counseling was provided, and medical referral was offered by organizing an appointment at a clinic belonging to a network of liver and infectious diseases public clinics set up at the beginning of the survey.

Participating Greek Roma settlements were visited several times to disseminate blood test results and to provide individual counseling and offer/organize referral when necessary. The whole procedure was set up carefully to ensure confidentiality of results. Migrant participants were called, and an appointment was arranged to disseminate blood test results and offer/organize referral for those with positive results. For those who did not respond to several attempts, an effort was made to get in contact with them through their communities or the collaborating NGOs. The turnaround time of results was around 3 months for the general population and 2 months for migrants and Greek Roma.

### Hepatitis B Virus Vaccination

HBV susceptible individuals from Greek Roma and migrants’ communities were offered the anti-HBV vaccine. To increase adherence to vaccination, a short vaccination scheme (first dose: day 0; second dose: 4 weeks; third dose: 16 weeks) was adopted (Multimedia Appendix 2). All susceptible individuals were informed, and the vaccine was offered to those who agreed and had no contradictions (ie, serious allergy to the components of the vaccine and acute febrile illness). Vaccination was performed in the participating settlements for Greek Roma and in the collaborating NGOs polyclinics for migrants.

### Study Promotion and Awareness Activities

Before study initiation, study dissemination/promotion activities took place as described above. After field study completion, awareness activities were implemented with an aim to improve the knowledge level of HBV, HCV, and HIV infections and to promote safe sexual behaviors. Population-specific awareness models were developed and implemented. All activities took place after field study completion to allow us to evaluate their effectiveness.

Population-specific brochures on transmission routes, testing, and treatment opportunities were developed. Greek Roma brochures were based on simple illustrative sketches (provided by the Spanish NGO Salud Entre Culturas) with minimal text, whereas the ones for migrants combined illustrative drawing (created pro bono by the painter Venetia Psara) with minimal text in 9 languages (Greek, English, French, Albanian, Romanian, Farsi, Urdu, Arabic, and Bengali).

For the general population, awareness actions included: (1) television and radio spots design and creation, once approved by the National Council for Radio and Television, these were distributed to all TV channels and radio stations; the main idea was around the concept *you cannot see/hear it, but it exists*; (2) informative events organized in collaboration with participating municipalities; (3) in some regions, events in high school were organized in collaboration with corresponding municipalities and school directors.

Actions in Greek Roma took place in the participating settlements and involved all community. They included (1) projection of specially created audiovisual material (short video) on hygiene behaviors, (2) distribution of usable items (wristbands with a message, condoms, etc), (3) implementation of experiential games targeting stereotype elimination and perceptions and increasing knowledge. Actions for migrants included (1) disclosure of usable items (identity card-certificate card with logo, message-wristbands, key chains, and condoms) and (2) information events in both Greek and English at the Doctors of the World Greek delegation and PRAKSIS, as well as in migrant communities. Information events were also organized for migrants with drug addiction in at the KETHEA organization and for homeless migrants at the Municipality of Athens. Where necessary, interpreters were present to relay information in the participants’ native language (eg, Ukrainian).

### Assessment of Awareness Activities

The effectiveness of awareness actions was evaluated in the general and Greek Roma populations. The high mobility of migrants during the field study period (2015-2016) made it difficult, if not impossible, to reach the participants after all field study (including vaccination) had been completed; thus, the effectiveness of the awareness actions was not evaluated in this population. A questionnaire was developed that included the following sections: (1) basic demographic information; (2) all questions about knowledge and attitudes on infectious diseases under investigation that were included in the main study; and (3) questions evaluating the experience and satisfaction of participants from all stages of the study. Of the initial sample, 10% (ie, 600 individuals) of the general population was selected via systematic stratified sampling; participants in each region were ranked in ascending order based on their barcode, and 1 out of every 10 participants was selected. To ensure randomness and representativeness of the evaluation sample, the starting point varied by region: in one region, the selection procedure started from the smallest barcode, in the next from the second smallest and so on.

In the Greek Roma population, awareness actions were evaluated by visiting the selected settlements. A total sample of 100 individuals, equally distributed among the regions, was selected, that is, 25 individuals from each of the 4 regions participated in the evaluation study. The same procedure as for the general population was applied to select the subsample, although 1 every 5 individuals of the initial sample was selected.

The effectiveness of awareness activities was assessed by comparing the knowledge and attitudes score (derived by relevant questions) before and after the implementation of the awareness activities in the subsample of the general and Greek Roma study populations.

### Cost

The total study implementation cost was around €772,000 (about €108 per participant). Total cost was distributed as 64% for personnel (academic and research); 20% for reagents; 5.2% for blood sample collection materials, transfer, and storage; 6.8% for study promotion and awareness activities materials; 2.7% for HBV vaccines; and 1.3% for portable computers and/or tablets for interviewing.

### Statistical Analysis

The general population survey had a complex study design, with varying sampling fractions across regions. Thus, for the statistical analysis, sampling weights, being reciprocal of the probability of selection, will be applied. In addition, to adjust for potential discrepancies between the sample age and sex distribution and that of the reference population recorded in the 2011 Census, poststratification weights should be applied. As not all interviewed individuals provided blood samples, inverse probability weighting or multiple imputation methods could be applied to adjust for potential selection bias in all analysis involving the blood test results. A more detailed description of the statistical analysis for the general population survey is provided elsewhere [27].

The lack of sampling frame for vulnerable populations (Greek Roma and migrants) leads to a conventional sample. Initial analysis of Greek Roma and migrant data will be crude, ignoring deviations from the initially desired distributions. In the second stage, weighted analysis will be applied to adjust for such deviations.

Statistical analysis of the data is underway, and the first results are expected to be submitted during 2020. Questionnaire data will be combined with blood test results; the statistical analysis will focus on (1) prevalence estimates and treatment cascade of the investigated infections in the 3 study populations; (2) testing frequency and associated factors; (3) knowledge level and its determinants; (4) assessment of awareness-raising activities; and (5) access to health care and its determinants. For vulnerable populations, living conditions will also be investigated.

## Results

### Distribution of Study Samples

In total, 6006 individuals from the general population, 534 Greek Roma, and 612 migrants were recruited. The overall response rate for interviewing in the general population was 72.0%. The distribution of the final achieved sample compared with the planned one for Greek Roma is presented in Table 1 and for the migrant population in Table 2. Compared to the planned sample, settlements with houses and settlements with houses and shacks were slightly underrepresented, and settlements with shacks were slightly overrepresented in the final achieved Greek Roma survey. In the migrant sample, Albanians and those who originated from Europe were to some degree underrepresented, and those from Africa and Asia were overrepresented in the final achieved sample compared with the planned sample.

### Demographic Characteristics of Study Participants

From the 6006 individuals of the general population, 13 refused to provide their age, and as this variable is necessary to compute poststratification weights, all results provided were restricted to the 5993 participants with available age. Descriptive characteristics of the 3 study populations are presented in Table 3. After applying weighting, sex distribution was balanced in the general population sample. Compared with men, the proportion of women was higher in the Greek Roma sample and lower in the migrant sample. As expected, the Greek Roma and migrant study populations were substantially younger than the general population. Education level was relatively high in the general population and in migrants with about 23.6% (1222) from the general population and 12.4% (76) of migrants having a university or higher educational level. On the contrary, the majority (50.6%, 270) of the Greek Roma were practically illiterate (ie, not completed primary school). For all 3 populations, most of the participants were married or in cohabitation (Table 3). Reflecting the consequences of the financial crisis, the household income was low in all study populations, with family income below €900 per month for about 39.7% (2401) of the general population; around 26.6% (142) of Greek Roma reported no income at all, whereas personal income was less than €350 per month for 58.7% (359) of the migrants. The unemployment rate was high in all populations. The estimated unemployment rate (95% CI) for those over productive ages (≤65 years) in the general population was 28.7% (26.9%-30.5%). The unemployment rate was 59.0% (561) in migrants, whereas it was above 36.5% (195) in the Greek Roma population.

In the general population sample, among those with reported country of birth (N=5907), about 11.1% (580) were born in a country other than Greece/Cyprus, a result in line with the ELSTAT report from the 2011 Census. In the migrant survey, around 46.1% (282) described themselves as documented migrants, 24.4% (180) as asylum seekers or on refugee status, and 21.7% (133) as undocumented migrants. Only 2.8% (17) did not reveal their migrant status.

**Figure 1:**
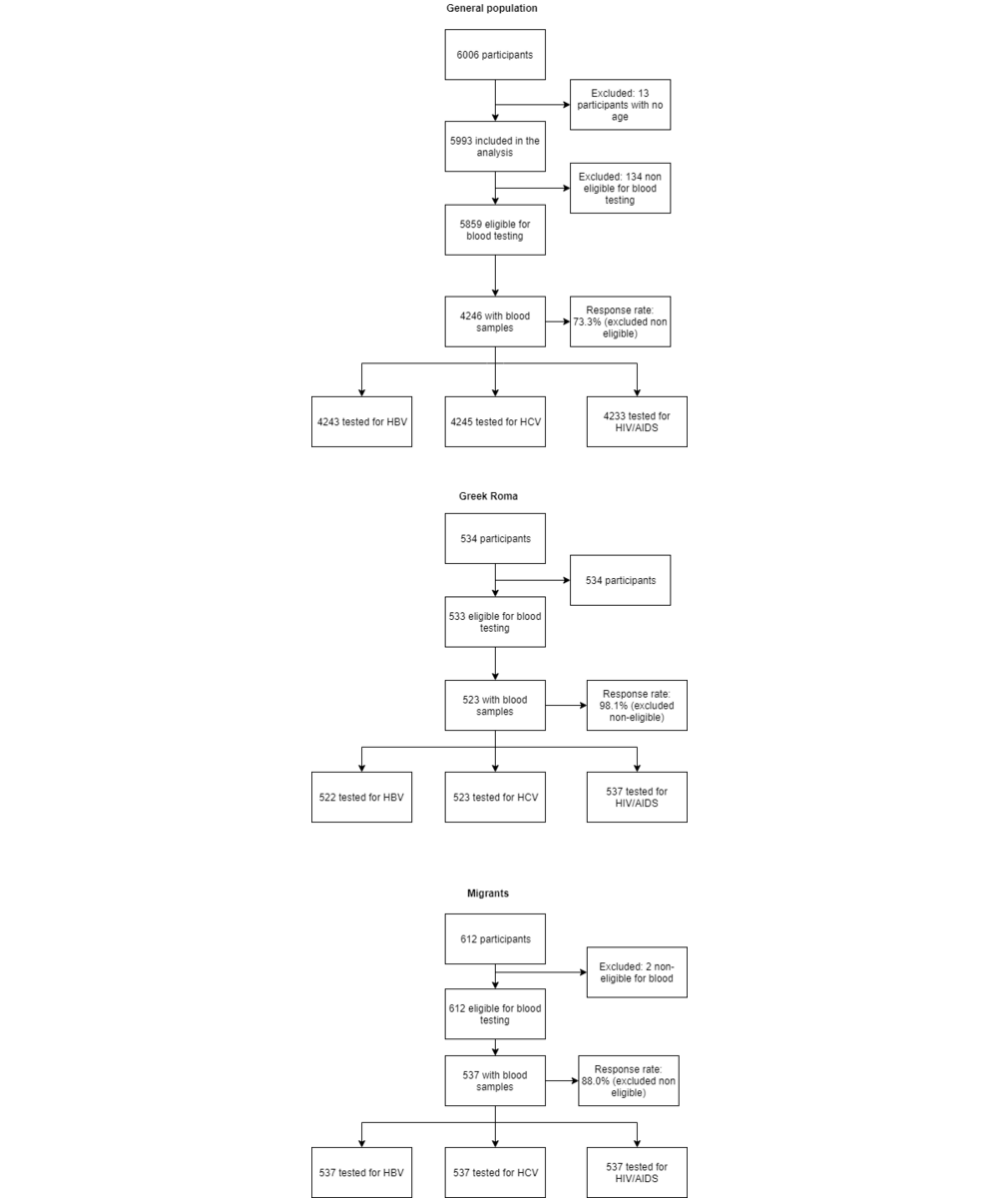
Data flowchart of the 3 studied populations (general population, Greek Roma, and migrants). HBV: hepatitis B virus. HCV: hepatitis C virus.

Among interviewed participants from the general population, 70.7% (N=4245) had serological tests, but 134 individuals (2.3%) had reasons to be excluded from blood sample collection (collaboration inability, pregnancy, anemia, and feeling faint); thus, among the eligible individuals, the response rate was 72.5% (4245/5859). In more detail, 4243 individuals were tested for HBV, 4245 for HCV, and 4233 for HIV/AIDS (Figure 1). The response rate was higher for Greek Roma reaching almost 98.0% (522/534) and 87.7% (537/612) for migrants. The numbers of Greek Roma and migrants tested for HBV, HCV, or HIV are presented in Figure 1.

**Table 3:** Demographic characteristics of study participants in the 3 study populations (general population, Greek Roma, and migrants).

Demographic characteristics			General population^a^		Greek Roma		Migrants
**Gender, n (%)**							
	Male	2546 (48.5)		247 (46.3)		340 (55.6)	
	Female	3447 (51.5)		287 (53.7)		271 (44.3)	
	Unknown	0 (0.0)		0 (0.0)		1 (0.2)	
Age (years), median (IQR)			47.7 (34-64)		35.0 (25-48)		36.9 (29.8-46.4)
**Educational level, n (%)**							
	No school	0 (0.0)		270 (50.6)		46 (7.5)	
	Up to primary	2114 (28.9)		202 (37.8)		105 (17.2)	
	Up to secondary or postsecondary	2575 (46.2)		48 (9.0)		369 (60.3)	
	University or higher	1222 (23.6)		0 (0.0)		76 (12.4)	
	Other/unknown	82 (1.3)		14 (2.6)		16 (2.6)	
**Family status, n (%)**							
	Married or in cohabitation	3936 (61.0)		422 (79.0)		382 (62.4)	
	Single	1995 (38.0)		111 (20.8)		226 (36.9)	
	Unknown/no answer	62 (1.0)		1 (0.2)		4 (0.7)	
**Legal status (for migrants only), n (%)**							
	With legal papers	N/A^b^		N/A		282 (46.1)	
	Asylum seeker/refugee status/humanitarian status	N/A		N/A		180 (29.4)	
	Without legal papers	N/A		N/A		133 (21.7)	
	Unknown	N/A		N/A		17 (2.8)	
**Household income/month^c^, n (%)**							
	Up to 900€^d^/no income^e^/up to 350€^f^	2401 (39.7)		142 (26.6)		359 (58.7)	
	900€-1700€^d^/<450€^e^/351-700€^f^	1658 (28.1)		305 (57.1)		96 (15.7)	
	>1700€^d^/≈450€^e^/701-900€^f^	606 (10.9)		63 (11.8)		14 (2.3)	
	—^d^/>450€^e^/>900€^f^	N/A		21 (3.9)		6 (1.0)	
	No answer	1328 (21.4)		3 (0.6)		137 (22.4)	
**Employment status, n (%)**							
	Employed	2088 (38.7)		140 (26.2)		160 (26.1)	
	Retired/household	2642 (35.4)		163 (30.5)		76 (12.4)	
	Unemployment	784 (15.3)		195 (36.5)		361 (59.0)	
	Other/unknown	479 (10.6)		36 (6.7)		15 (2.5)	
**Country of birth, n (%)**							
	Greece/Cyprus	5327 (88.9)		534 (100.0)		N/A	
	Balkans	294 (4.6)		N/A		221 (36.2)	
	East Europe/former Soviet Union	89 (1.6)		N/A		57 (9.7)	
	West Europe/Australia/Americas	101 (1.8)		N/A		1(0.2)	
	East/Central Asia	34 (0.7)		N/A		3 (0.5)	
	Middle East and North Africa	27 (0.5)		N/A		78 (12.8)	
	South Asia	17 (0.3)		N/A		124 (20.2)	
	Sub-Saharan Africa	18 (0.3)		N/A		126 (20.8)	
	Unknown	86 (1.4)		N/A		N/A	
**Residence type (for Greek Roma only), n (%)**							
	Settlement with houses	N/A		165 (30.9)		N/A	
	Settlement with houses and shacks (mixed)	N/A		232 (43.4)		N/A	
	Shacks	N/A		137 (25.7)		N/A	

^a^Weighted percentages.

^b^N/A: not applicable.

^c^For migrants, income refers to personal income, whereas for the other study populations, it refers to family income.

^d^General population.

^e^Greek Roma.

^f^Migrants.

## Discussion

### Strengths and Limitations

Hprolipsis is the first nationwide epidemiological study on infectious diseases HBV, HCV, and HIV. It was conducted not only in a random sample of the general population but also in two hard-to-reach populations, Greek Roma and migrants. In total, 6006, 534, and 612 from the general population, Greek Roma, and migrants, respectively, were recruited. Of those from the general population, 4243 were tested for HBV, 4245 for HCV, and 4233 for HIV/AIDS. The count for those tested for HBV, HCV, and HIV/AIDS in Greek Roma was 522, 523, and 523, respectively, and the count for those tested for HBV, HCV, and HIV/AIDS in migrants was 537, 537, and 537, respectively. Hprolipsis has 2 main advantages: (1) it combines extensive questionnaire data on several health indices with blood sample test results and (2) although one of its aims was to estimate the prevalence of HBV, HCV, and HIV infections in the general adult population and in the 2 vulnerable populations, it was not only a research study but also an integrated program providing individual counseling, referral to all individuals who tested positive, and vaccination in vulnerable populations. The study was also involved in designing and implementing population-specific awareness actions, the success of which was evaluated by comparing the level of knowledge on the 3 investigated infections before and after awareness actions took place.

The expected benefits are multifaceted. Greece will be able to provide valid estimates of the prevalence of HBV and HCV infections in the general population, Greek Roma, and migrants. Such data, currently lacking, are particularly useful in an era where global efforts to control and ultimately eliminate these infections are being taken. Indeed, preliminary results on HCV prevalence, presented at international conferences [31], were used as the best currently available estimates on which the National Plan for eliminating HCV infection was based. In addition, data on testing, diagnosis, and treatment rates, overall and by risk group, region and sex, will provide the necessary evidence to construct disease-specific cascades of care and to identify potential inequalities. Dissemination of study results to health policy makers, relevant NGOs, and local authorities will help them to set up targeted prevention measures and to adjust health policies. Hprolipsis prevalence estimates can be used as baseline values to assess future changes and future disease burden. When combined with data on the use of health services and medicines in health economic models, future health needs and costs, necessary for public health policy planning, can be estimated.

Conducting a survey in migrant and in particular in Greek Roma populations is not an easy task (eg, [32-34]), and it becomes even more difficult when infectious diseases such as viral hepatitis and HIV are of main interest, owing to their stigmatization. Few such studies have been conducted worldwide (eg, [3,20,34-36]). Several issues, with the most important one being the establishment of trust relationships with target populations, must be considered. Despite the moderate deviations of the final sample, as compared with the planned one, within Hprolipsis, we managed to successfully complete the survey in Greek Roma and migrants. Conducting this study provided us with valuable experience. Dissemination of our study materials (study design, SOPs, guide on how to set up trust relationships, and lessons learned) will provide useful information for future studies on difficult-to-reach populations.

Data on the level of knowledge and misconceptions of HBV, HCV, and HIV routes of transmission and treatment opportunities as well as on prejudices against individuals testing positive are lacking in Greece. Such data, however, are necessary to design and implement awareness campaigns. In addition, knowledge, misconceptions and prejudices may differ by risk population. In Hprolipsis, we assessed the level of knowledge, misunderstanding, and prejudice in all 3 study populations. The analyses of these data will highlight the population-specific gaps in knowledge. Successful awareness campaigns must consider the specific cultural and social characteristics of the target populations. In Hprolipsis, populations-specific awareness-raising campaigns were designed and implemented, and their effectiveness was evaluated. Experience gained through this procedure, when shared with relevant governmental organizations and NGOs, could provide valuable information for designing future campaigns.

Finally, study participants gained numerous benefits. They were provided with HBV, HCV, and HIV blood test results, received personalized advice, and were personally informed about the routes of transmission and therapy opportunities for these infections. They also had the opportunity to discuss other health issues with the study physicians, if they wanted. In addition, previously undiagnosed cases took advantage of their early diagnosis, personal counseling, and referral to specialized clinics. Greek Roma people living in the selected settlements additionally benefited from all other provided services. Greek Roma and migrants susceptible to HBV also had the opportunity to be vaccinated for HBV.

Despite the significant advantages of the study, it also had some limitations. One major limitation was the lack of sampling frame for Greek Roma and migrants. Thus, a convenience sample was chosen. Despite our efforts to review all available data and assess sex, age, and ethnicity distribution and to imitate this distribution in our sample, representativeness cannot be assumed. However, in the Greek Roma survey, the sample was drawn from the regions where excessive number of Greek Roma live; across each selected region, representative settlements of all 3 types (homes, mixed, and shacks) were chosen to ensure that there will be enough people from each settlement type to enable investigation of the effects of the living conditions on health indices and that less privileged settlements were well represented in the sample. To increase representativeness, migrants were recruited from several sources, as suggested [22,28]. In the migrants’ survey, we tried to catch the contemporary migrant flows. On the basis of all available data review, new arrivals mainly originate from Eastern European countries, Africa and, more recently, from Asia owing to the war in Syria. Those migrants are likely to be less privileged and with the most unmet needs. In our sample, a substantial proportion originated from Africa, Asia, and East European countries. In the general population survey, about 10% of the total sample was born in a country other than Greece (6% in a Balkan country, mainly Albania), in line with 2011 Census reports and justifying our decision to underrepresent Albanians in the migrants’ survey. The sampling methodology for the general population ensures representativeness. However, the door-to-door approach that was adopted to recruit study participants, although quite common for conducting health screening, has some restrictions as well, mainly associated with increased costs, reducing response rate, and interviewers’ safety [27,37,38]. Despite these limitations, the overall response rate in our study was quite high (72%).

Another limitation was that the migrants’ field study was conducted during 2015 and 2016, a period of high migrant mobility. Although this could not be predicted at the study design phase, it had some serious side effects, the most important of which was that, despite multiple attempts, some participants could not be easily traced to provide them with test results, counseling, and referral or vaccination if needed. Therefore, for future studies in displaced people, fourth-generation rapid tests for HBV, HCV, and/or HIV should be recommended. Despite their higher cost, rapid tests minimize the time needed from testing to diagnosis, counseling, and referral. Although this course of action would be obvious nowadays, blood sample testing was the usual approach at the time this study was designed. Similar issues were raised during vaccination. Although we chose a medium/short vaccination scheme (0, 4, and 16 weeks) as more appropriate for vulnerable populations, several eligible individuals, mainly migrants, failed to complete their vaccine scheme. Alternative schemes must be explored in future studies. Faster schemes (eg, 0, 1, and 3 weeks with the first dose given to all people irrespective of being susceptible), although they require a booster dose at 1 year to increase rates of immune coverage, may be preferable. In general, vaccination schemes must be adjusted to the specific characteristics of the target population.

Finally, the required sample size for the general population was estimated based on HCV prevalence, which is expected to be lower than HBV prevalence. However, HIV infection in the general population is expected to be much lower than that of HCV (around 0.13%), and thus, estimates of HIV prevalence are expected to be of low precision.

### Conclusions

In conclusion, the Hprolipsis results will increase our understanding on health needs and burden of infectious diseases not only in the general population but also in Greek Roma people and migrants. Policy makers, NGOs, and Greek Roma and migrant communities can rely on the Hprolipsis results for planning population-targeted interventions. The implementation of this integrated program was a great experience, providing useful recommendations for future studies, particularly in vulnerable populations. However, to have a complete picture of the total burden of the investigated diseases in Greece, similar studies in other vulnerable populations such as homeless individuals, prisoners, and sex workers are needed.

## Appendix

Multimedia Appendix 1 Detailed survey sections and questionnaire items.

Multimedia Appendix 2 Immunization card.

## Abbreviations

anti-HCV: Hepatitis C Antibody
ELSTAT: Hellenic Statistical Authority
EMENO: National Survey of Morbidity and Risk Factors
HBsAg: Hepatitis B surface Antigen
HBV: hepatitis B virus
HCDCP: Hellenic Centre for Disease Control and Prevention
HCV: hepatitis C virus
NGO: nongovernmental organization
NKUA: National and Kapodistrian University of Athens
PICF: participant information and consent form
SC: steering committee
SOP: standardized operating procedure
