# Supplementary material for: Design and Development of a Viral Hepatitis and HIV Infection Screening Program (Hprolipsis) for the General, Greek Roma, and Migrant Populations of Greece: Protocol for Three Cross-Sectional Health Examination Surveys
Source: JMIR Res Protoc. 2020 Jan 31;9(1):e13578. doi: 10.2196/13578 (PMC7055811; doi:10.2196/13578)
Supplement: Multimedia Appendix 1 [file resprot_v9i1e13578_app1.docx]

| Survey sections | Questionnaire items |
| --- | --- |
| Section | Details and items (numbered) |
| Administrative information | Participant barcode, place of interview, address, home address, e-mail address, phone number |
| Basic information: Sociodemographic and personal characteristics | People living in household (sex, adults/children), sex, date of birth (or age), marital status, cohabitation with the partner, children existence, country of birth, citizenship/s, nationality, educational level, years at school, current working status, paid work, activity sector, insurance, household income, household financial difficulties |
| Health status | General health, chronic diseases |
| Health system | Health system usage, health services satisfaction, health costs, medicine use, factors associated with health (alcohol consumption, nutritional insecurity, smoking, passive smoking |
| Knowledge on hepatitis B/C | 1. A person with hepatitis B/C can transmit it to someone else 2. A person with hepatitis B/C can transmit it to someone else 3. If someone has hepatitis B/C but looks and feels healthy then she/he cannot transmit the virus to another person 4. Hepatitis B/C can lead to cirrhosis or liver cancer 5. The only way to learn someone if he/she has hepatitis B/C is to do the corresponding test 6. There is a preventive vaccine for hepatitis B/C 7. There is treatment for hepatitis B/C |
| Knowledge on HIV | 1. HIV and AIDS are the same 2. If someone is HIV positive has symptoms 3. If someone has HIV/AIDS but looks and feels healthy, then she/he cannot transmit the virus to another person 4. HIV infection can cause a decline in body’s defense 5. There is a preventive vaccine for HIV/AIDS 6. The only way someone to learn if she/he is positive is to do the corresponding test 7. There is treatment for HIV/AIDS 8. (if you answered “true” in previous question) Treatment can cure the individual definitively from HIV infection |
| Transmitted modes of hepatitis B/C and HIV | 1. Transfusion from infected blood 2. Sexual intercourse without a condom 3. From mother to child 4. Daily social contact (shake hand, conversation) 5. By drinking or eating from the same utensils with someone infected 6. By using the same toilet, pool, sauna with someone infected 7. Kiss 8. Mosquito bite 9. Tattoo or body piercing 10. Intravenous drug use |
| Previous testing for hepatitis B/C and HIV infection |  |
| Risk assessment for hepatitis B/C and HIV infection | 1. Work: Contact with blood, blood products, syringes or needles 2. Medical history: (2.1) surgery with narcosis, (2.2.) endoscopy (gastroscopy, colonoscopy), (2.3.) blood transfusion (before 1992), (2.4.) hemodialysis (artificial kidney), (2.5) organ transplantation 3. Member of household diagnosed with hepatitis B/C 4. Ever diagnosed with sexually transmitted disease (eg gonorrhea, chlamydia, syphilis, genital herpes, wars) 5. Ever treated for sexually transmitted disease (eg gonorrhea, chlamydia, syphilis, genital herpes, warts) 6. Lifestyle-habits: (6.1.) tattoo or body piercing, (6.2.) drug injections, (6.3.) more than 5 new sexual partners during the last year |
| Sexual behavior | Lifetime number of sexual partners, usually usage of condom, reasons for using condom, condom use during last intercourse |
