# Supplementary material for: Design and Development of a Viral Hepatitis and HIV Infection Screening Program (Hprolipsis) for the General, Greek Roma, and Migrant Populations of Greece: Protocol for Three Cross-Sectional Health Examination Surveys
Source: JMIR Res Protoc. 2020 Jan 31;9(1):e13578. doi: 10.2196/13578 (PMC7055811; doi:10.2196/13578)

***
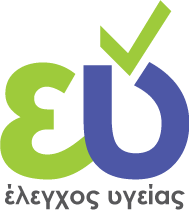

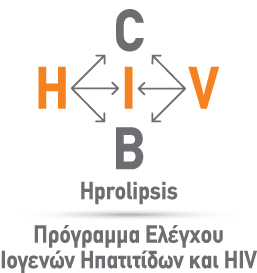
***

***IMMUNIZATION FOR HEPATTIS B***

| *barcode* | | *Initials:* | *Sex:* |
| --- | --- | --- | --- |
|  |  | *Date of birth:* | |
|  | ***planning*** | ***realization*** | |
| **1^st^ dose** | Date | Date-signature | |
| **2^nd^ dose** | Date | Date-signature | |
| **3^rd^ dose** | Date | Date-signature | |


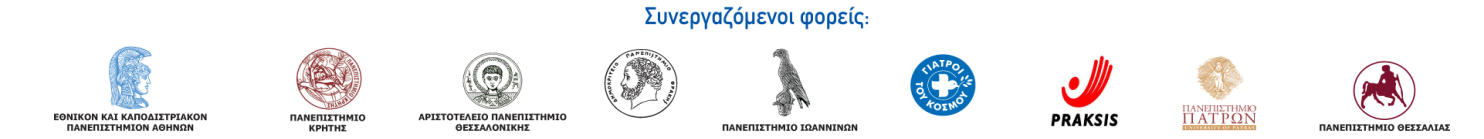

Supplement: Multimedia Appendix 2 [file resprot_v9i1e13578_app2.docx]
